# Book review: Human Rights in Global Health: Rights-Based Governance for a Globalizing World

**DOI:** 10.1186/s12992-019-0491-9

**Published:** 2019-07-23

**Authors:** Unni Gopinathan

**Affiliations:** 1 Oslo Group on Global Health Policy, Department of Community Medicine and Global Health & Centre for Global Health, Institute for Health and Society, Faculty of Medicine, University of Oslo, Oslo, Norway; 2 Cluster for Global Health, Division for Health Services, Norwegian Institute of Public Health, Oslo, Norway

**Keywords:** Global health governance, Global governance for health, Human rights, International law, Social determinants of health

## Abstract

This is a review of the book “Human Rights in Global Health: Rights-Based Governance for a Globalizing World” edited by Benjamin Mason Meier and Lawrence O. Gostin.

## Main text

Institutions of global governance play a critical role in diffusing norms and influencing the behaviour of states and non-state actors [1, 2]. Previously, scholars have examined how policies promoted by global governance institutions impact human rights [3], how these institutions can advance human rights norms [4–6], and how organizational culture influences the adoption of policies promoting human rights [7]. A broad range of global governance institutions influence areas that intersect with health and human rights. Yet no comprehensive comparative assessment of how these institutions have operationalized health-related human rights have been produced.

Against this background, the volume ‘Human Rights in Global Health: Rights-Based Governance for a Globalizing World’, edited by Benjamin Mason Meier and Lawrence O. Gostin, brings together an impressive group of academics, individuals from UN and international civil society organizations, and policymakers to make a far-reaching contribution to close this analytical gap [8].

Following a clear and logical structure, the book’s introductory chapter and first section clarify its expansive focus on the underlying social, economic and political causes (e.g. ‘determinants’) of health and illness, thereby broadening the scope of global institutions beyond those we commonly consider to do ‘global health’ work. The next three chapters are about the World Health Organization (WHO)—the world’s normative authority on global health. These chapters give insights into how WHO at critical junctures in its history, from the drafting of its constitution to facing the HIV/AIDS pandemic, has struggled to reconcile a technical role focused on standard-setting with its constitutional authority to advance human rights norms. Moreover, these provide forward-looking reflections suggesting how WHO can assume a greater role in advancing rights-based approaches.

Going beyond WHO, the subsequent three sections examine the institutional mandate and the evolution of various global institutions, and how these institutions have engaged with and operationalized the right to health through their policies, programs and practices. Among the book’s many strengths are that these chapters broadly follow a unifying framework that enables comparison of the approaches of these institutions. This framework introduces the reader to:

- the historical origins of each institution
- the relationship between the institutions’ mission, and international conventions and soft law instruments such as declarations and codes of practice, and how this relationship form the foundations of their human rights engagement:
- how human rights have been mainstreamed and advanced through programmes and policies; and
- the institutional determinants, e.g. the distinct features of each institution that facilitate or impede organizational efforts to advance human rights

The chapters introduce how some institutions, such as UNAIDS, were borne out of a health and human rights imperative [9]. Others, such as UNICEF, have seen an opportunity to transform their institutional authority through international human rights treaties (in UNICEF's case the Convention on the Rights of the Child). In stark contrast are the multilateral institutions of global economic governance, particularly the World Bank and the World Trade Organization, representing more contested spaces where promotion of health-related human rights have clashed with policies dictated by prevailing ideas and ideologies within the institutions. Accordingly, human rights have been modified, distorted or resisted when interpreted and translated into institutional activities [10]. Yet even in the absence of a formal relationship with human rights law, these chapters demonstrate an ongoing evolution where institutional factors within these organizations are facilitating active engagement with health and human rights considerations. The volume concludes with the editors analyzing generalizable themes and raising a research imperative: continued analysis of how the institutions that collectively constitute global governance for health can more forcefully promote health-related human rights.

Have any topics been missed in this volume? First, a separate chapter could perhaps have been devoted to the United Nations Development Programme (UNDP), given its history in invoking human rights in response to the HIV/AIDS epidemic [11] and more recently through its broader global health strategy [12]. Second, a chapter about an institution in global environmental governance would have further enriched the volume, given its ambitious focus on the underlying causes of health and the influences of issues such as climate change, air pollution and biological diversity on global health [13–15]. Finally, in their concluding chapters the editors highlight civil society participation as crucial for human rights mainstreaming. While some chapters (such as the one about UNFPA) describe in great detail how global institutions are facilitating civil society involvement, the volume could perhaps have made the assessment of civil society participation a more visible cross-cutting theme across chapters.

Notwithstanding these observations, what stands out in this book is the breadth and detail by which institutions have been analyzed and compared. This volume is a timely contribution to the global health discourse. The sustainable development goals (SDGs) have stimulated increased attention towards the broader determinants of health [16]. Yet major global health policy processes still tend to fall short of involving the broad range of global governance institutions that, in this volume’s own words, “exercise their institutional mandate in ways that influence public health” [8]. A case in point is the ongoing process of developing a Global Action Plan for healthy lives and well-being for all [17]. Coined as a “historic commitment to unite for health”, the process recognizes that accelerating progress on SDG3 “requires unified efforts to address the determinants of health and the health inequities or disparities such determinants perpetuate”. It is impressive that this process has 12 signatory global agencies from health, development and humanitarian areas [17]; however, these together do not fully reflect—again using the words of the volume—the “multi-sectoral array of determinants of health”, with agencies such as FAO, ILO, UNEP and UNHCR missing from the picture. This volume edited by Mason Meier and Gostin presents a compelling case for why the global health discourse on a consistent basis should involve a broader range of global governance institutions. Moreover, the volume engenders hope that global institutions gradually can move towards human rights as a shared normative framework going forward. Everyone concerned with reinvigorating efforts to address the broader determinants of health and reduce health inequities will benefit from reading this book.

## Data Availability

N/A
